# Incidental Finding of Dextrocardia with Situs Inversus in a 59-Year-Old Man

**DOI:** 10.1155/2019/7107293

**Published:** 2019-12-01

**Authors:** Emmanuel Kobina Mesi Edzie, Klenam Dzefi-Tettey, Obed Cudjoe, Philip Narteh Gorleku, Patrick Adu

**Affiliations:** ^1^Department of Medical Imaging, School of Medical Sciences, College of Health and Allied Sciences, University of Cape Coast, Cape Coast, Ghana; ^2^Department of Imaging Technology and Sonography, School of Allied Health sciences, College of Health and Allied Sciences, University of Cape Coast, Cape Coast, Ghana; ^3^Department of Microbiology and Immunology, School of Medical Sciences, College of Health and Allied Sciences, University of Cape Coast, Cape Coast, Ghana; ^4^Department of Radiology, Korle-Bu Teaching Hospital, Accra, Ghana; ^5^Department of Medical Laboratory Sciences, School of Allied Health Sciences, College of Health and Allied Sciences, University of Cape Coast, Cape Coast, Ghana

## Abstract

Dextrocardia with situs inversus is a rare congenital anomaly, which is characterized by right-sided heart apex and inversely rotated visceral organs of the abdomen. We report an unusual case of dextrocardia with situs inversus in a 59-year-old man, referred for a pelvic ultrasound scan because of symptoms of lower urinary tract obstruction and after a fairly normal prostate specific antigen (PSA) value. A diagnosis of enlarged prostate gland with a prominent median lobe and significant residual urine volume was made, which necessitated the examination of the kidneys for hydronephrosis, resulting in the incidental finding of situs inversus. On further investigation, the diagnosis of dextrocardia with situs inversus was made. Physicians should look out for this anomaly primarily because it may be associated with other conditions like primary ciliary dyskinesia so appropriate interventions are offered to reduce morbidities and mortality.

## 1. Introduction

Dextrocardia with situs inversus (situs inversus totalis) is a very rare congenital defect characterized by reversal of the position of the heart to the right side of the thoracic cavity along with all inversely rotated visceral organs (mirror image) [1, 2]. It is a rare anomaly with incidence rate of 1/10,000 live births [1, 3]. The exact cause of dextrocardia is also unknown. However, it has been linked with a number of factors which include autosomal recessive gene inheritance, maternal diabetes, cocaine use and conjoined twinning along with equal ratio seen in both gender [4–6]. Individuals with situs inversus are unaware of their unusual congenital anomaly until they seek medical attention for totally unrelated conditions [7]. Individuals with dextrocardia and situs inversus may have associated congenital heart malformations, primary ciliary dyskinesia, or splenic malformations [8, 9]. We report an unusual case of dextrocardia with situs inversus in a 59-year-old man with an enlarged prostate gland with a prominent median lobe and significant residual urine volume, the first case to be reported in our region.

## 2. Case Report

A 59-year-old police officer, presented at the outpatient clinic with dysuria, frequency, hesitancy and a fairly normal PSA value of 4.13 ng/dl. Examination of his cardiovascular system revealed a normal pulse rate of 72 beats per minute and his blood pressure was 113/70 mmHg. Patient did not have any cardiopulmonary symptoms like chronic coughs and nasal congestion. His chest was clinically clear. Following a negative urine culture, the patient was sent for a prostate ultrasound scan, which revealed an enlarged prostate gland with a prominent median lobe and a residual urine volume of 91.79 cm^3^. This observation necessitated a kidney ultrasound scan examination to check for hydronephrosis.

**Figure 1:**
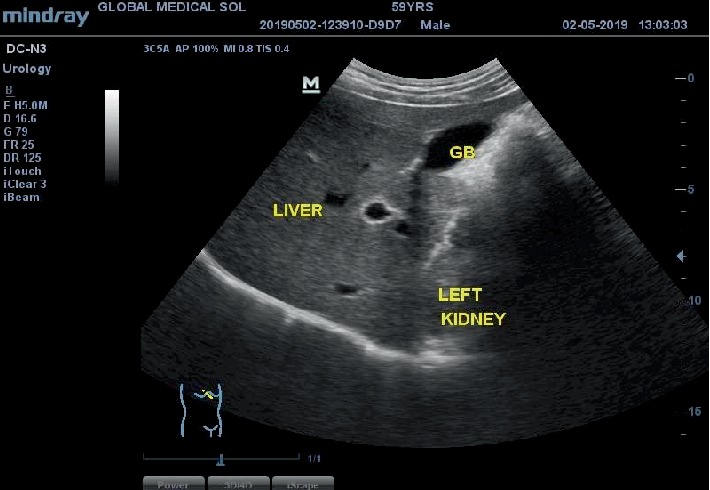
Ultrasound image of the epigastric region in longitudinal plane. This shows a left-sided liver and gallbladder instead of its normal right-sided anatomical position.

During the examination of the kidneys, the ultrasound scan showed the liver and gall bladder in the left hypochondrium as shown in Figure 1.

**Figure 2:**
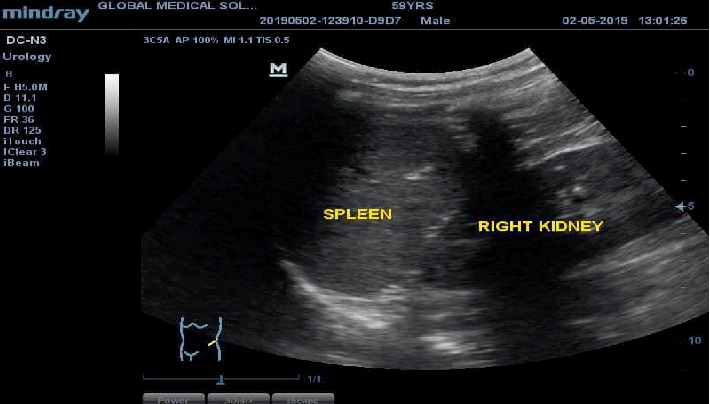
Sonogram of the right hypochondrium in longitudinal plane. The spleen is in the right upper quadrant under the right hemidiaphragm adjacent to the right kidney.

The spleen was also seen in the right hypochondrium by the right kidney instead of it's usual left-sided position, as depicted in Figure 2.

**Figure 3:**
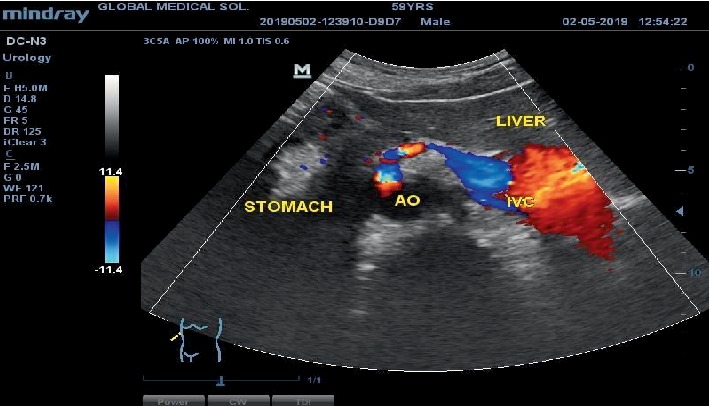
Colour Doppler ultrasound image of the epigastrium in transverse plane. This demonstrates a right-sided abdominal aorta (AO) and left sided inferior vena cava (IVC). Also showing a right-sided stomach and a left-sided liver, which is the reverse of their normal anatomical positions.

Further examination of the abdomen with colour Doppler visualised the abdominal aorta on the right toward the transposed stomach and the inferior vena cava on the left side toward the transposed liver as shown in Figure 3.

**Figure 4:**
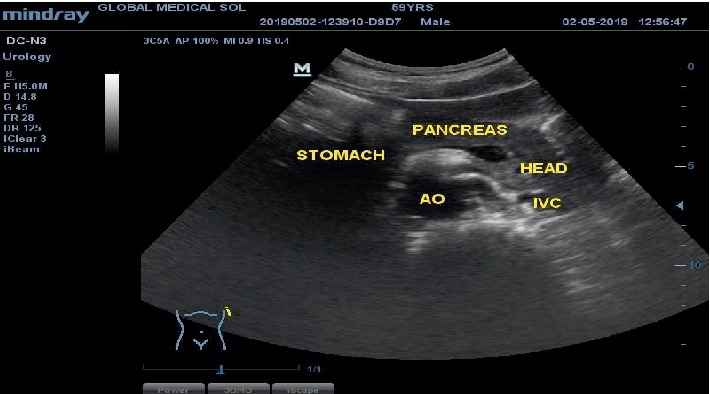
Transverse sonogram without Colour Doppler of the epigastrium. The head of the pancreas on the left side and a gas filled stomach on the right side. Abdominal aorta (AO) on the right side and inferior vena cava (IVC) on the left.

The head of the pancreas was also seen on the left side of the abdomen departing from the usual anatomy where it is on the right side as depicted in Figure 4.

**Figure 5:**
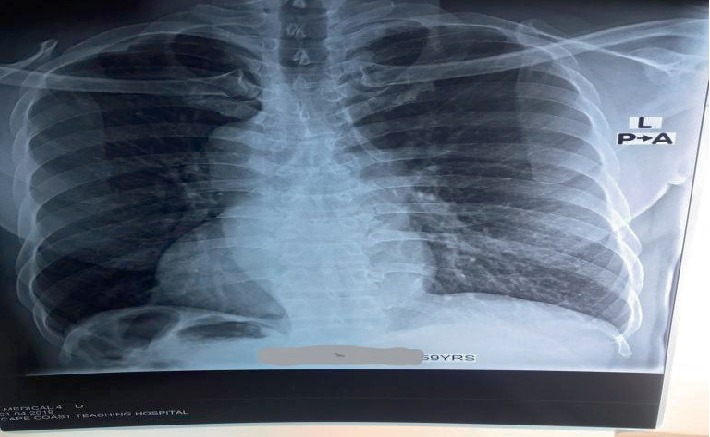
Frontal chest radiograph. It shows the apex of the heart pointing to the right, ascending and arch of the aorta on the right, gastric air bubble and splenic flexure both on the right side under the diaphragm opposite of the normal anatomical findings.

The findings were explained to the patient and his consent was sought for a chest radiograph at no cost to him. This revealed a normal lung fields with mild rotation of the patient towards the right and a dextrocardia with ascending aorta and the arch of the aorta all on the right side of the chest. The gastric air bubble was also seen below the right hemidiaphragm, large bowel gas pattern with haustrations likely the splenic flexure was also noted in the right hypochondrial region. The left hemidiaphragm was higher than the right and no gastric air bubble was seen under the left hemidiaphragm as shown in Figure 5, which is usually not the case in normal radiological anatomy.

The patient's unusual anatomy was explained thoroughly to him, he was counselled appropriately, reassured, and had his fears allayed. Permission and informed written consent were obtained for further investigations like echocardiography and barium/gastrographin swallow spot image. Echocardiogram also demonstrated dextrocardia with ascending aorta and arch of the aorta originating from the right. No other cardiac abnormalities detected.

**Figure 6:**
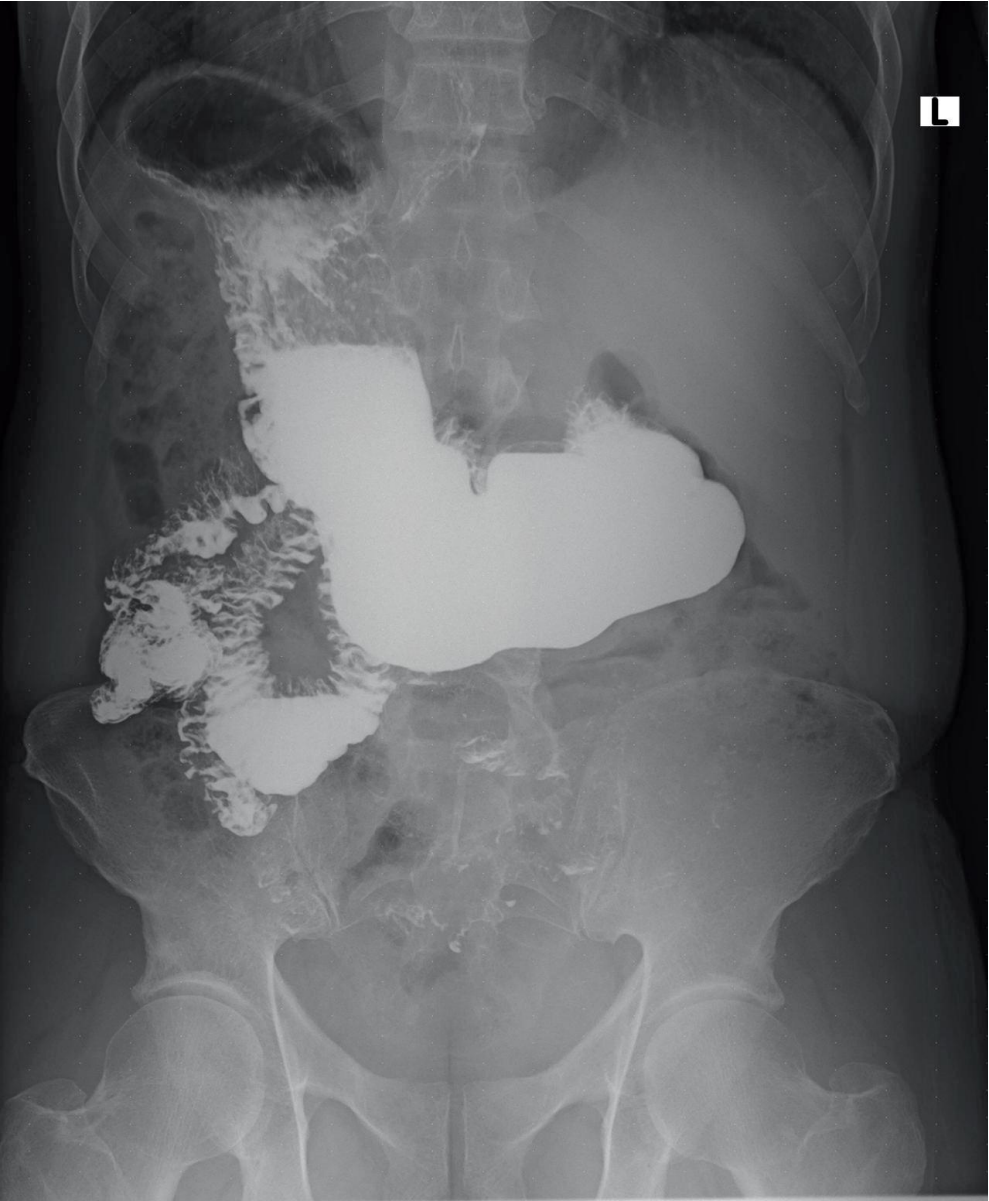
Barium sulphate meal spot radiograph. This shows the stomach on the right under the right hemidiaphragm, a definite air-barium level under the right hemidiaphragm with the heavier barium inferiorly and gastric air superiorly.

As demonstrated in Figure 6, barium sulphate solution meal spot image clearly showed a definite right-sided stomach with air-barium level under the right hemidiaphragm, the heavier barium inferiorly and lighter gastric air superiorly and confirming the reversal of the stomach from the left hypochondrium to the right.

## 3. Consolidated Findings for this Case

There was transposition of the abdominal viscera with the liver, gallbladder, head of the pancreas, and the inferior vena cava normally located on the right side of the abdomen now seen on the left side and the left sided spleen, stomach, aorta, and the tail of the pancreas now visualized on the right side. Also the posteroanterior chest radiograph showed apex of the heart pointing to the right and the aortic knuckle and descending aorta on the right side rather than on the left side in the normal anatomy consistent with situs inversus totalis.

## 4. Discussion

Situs inversus totalis is a rare congenital anomaly. There is no known cause of dextrocardia, but maternal diabetes mellitus and cocaine use by the mother have been implicated. Genetic factors are also suspected, with an increased incidence seen in conjoined twins. Situs inversus totalis shows no racial and sex predilection [4–6]. Dextrocardia with situs inversus poses a considerable danger as it remains asymptomatic and normally remains undiagnosed unless diagnosed incidentally while investigating for another ailment. In our report, the diagnosis was made during investigation of cause of dysuria and symptoms of the lower urinary tract obstruction. The common congenital cardiac anomalies associated with dextrocardia with situs inversus are atrial situs solitus (93%), discordant AV connection (44%), and discordant Ventriculo-Atrial (VA) connection (30%). Congenitally corrected Transposition of Great Arteries (TGA) occurs in less than 1% of all forms of congenital heart disease [10]. Certain congenital anomalies such as polysplenia (left isomerism)/asplenia (right isomerism) or Kartagener's syndrome often leading to infection of the paranasal sinuses and lungs) are known to occur [11]. About 25% of individuals with situs inversus have an association with primary ciliary dyskinesia. Situs inversus totalis with primary ciliary dyskinesia together known as Kartagener`s syndrome characterized by the triad of situs inversus, chronic sinusitis, and bronchiectasis [12]. However in this case, the patient was well without any history of sinusitis, chronic cough, and his chest was clinically clear.

Computer tomography (CT) remains the best imaging procedure for the definitive diagnosis of dextrocardia with situs inversus as CT scan provides an excellent anatomic detail. Magnetic resonance imaging (MRI) is reserved for patients with associated cardiac abnormalities [13, 14]. Electrocardiogram can however, confirm the medical diagnosis of the two forms of dextrocardia and also can show inversion of the electrical waves. This is considered one of the best diagnostic test options [2].

This case report goes to confirm that an individual can be born with situs inversus totalis and if not associated with any syndrome can remain asymptomatic and only incidentally diagnosed during radiological investigation for an unrelated disease. It is interesting to note that as radiologists of many years of working experience in general diagnostic radiology which includes the chest and abdominal imaging, this is the first time we have encountered this condition in our sub region.

## 5. Conclusion

An incidental finding of situs inversus totalis in a 59-year-old man is reported and the need for physicians to have high index of suspicion is highlighted due to it`s asymptomatic nature. This is the first case of this condition we are seeing in many years of our radiological practice in our region emphasizing the rarity of the condition. The misdiagnosis of situs inversus totalis can have dire consequences for the patient`s quality of life with chronic coughs and frequent respiratory tract infections if it is associated with primary ciliary dyskinesia. Hence the primary reason for practitioners and patients to know about this condition is to be in the position to offer the necessary and appropriate treatments for those with associated primary ciliary dyskinesia and for such patients to comply with treatments, in order to reduce associated long-term morbidities and ultimately mortality. Also since patients with this condition have reversal of anatomical structures, serious morbidity may occur during surgical interventions in a patient with an undiagnosed situs inversus totalis.

Routine medical examinations for employment or other reason must be taken seriously in which a case of dextrocardia will not be missed on a professionally taken chest radiograph.
